# Dying to Be Noticed: Epigenetic Regulation of Immunogenic Cell Death for Cancer Immunotherapy

**DOI:** 10.3389/fimmu.2018.00654

**Published:** 2018-04-03

**Authors:** Brianne Cruickshank, Michael Giacomantonio, Paola Marcato, Sherri McFarland, Jonathan Pol, Shashi Gujar

**Affiliations:** ^1^Department of Pathology, Dalhousie University, Halifax, NS, Canada; ^2^Department of Microbiology and Immunology, Dalhousie University, Halifax, NS, Canada; ^3^Department of Chemistry and Biochemistry, The University of North Carolina at Greensboro, Greensboro, NC, United States; ^4^Department of Chemistry, Acadia University, Wolfville, NS, Canada; ^5^Gustave Roussy Comprehensive Cancer Institute, Villejuif, France; ^6^INSERM, U1138, Paris, France; ^7^Equipe 11 labellisée par la Ligue Nationale contre le Cancer, Centre de Recherche des Cordeliers, Paris, France; ^8^Université Paris Descartes, Université Sorbonne Paris Cité, Paris, France; ^9^Université Pierre et Marie Curie, Paris, France; ^10^Department of Biology, Dalhousie University, Halifax, NS, Canada; ^11^Centre for Innovative and Collaborative Health Services Research, IWK Health Centre, Halifax, NS, Canada

**Keywords:** tumor microenvironment, immunogenic cell death, epigenetics, T cell immunity, cancer immunotherapy, immune evasion

## Abstract

Immunogenic cell death (ICD) activates both innate and adaptive arms of the immune system during apoptotic cancer cell death. With respect to cancer immunotherapy, the process of ICD elicits enhanced adjuvanticity and antigenicity from dying cancer cells and consequently, promotes the development of clinically desired antitumor immunity. Cancer ICD requires the presentation of various “hallmarks” of immunomodulation, which include the cell-surface translocation of calreticulin, production of type I interferons, and release of high-mobility group box-1 and ATP, which through their compatible actions induce an immune response against cancer cells. Interestingly, recent reports investigating the use of epigenetic modifying drugs as anticancer therapeutics have identified several connections to ICD hallmarks. Epigenetic modifiers have a direct effect on cell viability and appear to fundamentally change the immunogenic properties of cancer cells, by actively subverting tumor microenvironment-associated immunoevasion and aiding in the development of an antitumor immune response. In this review, we critically discuss the current evidence that identifies direct links between epigenetic modifications and ICD hallmarks, and put forward an otherwise poorly understood role for epigenetic drugs as ICD inducers. We further discuss potential therapeutic innovations that aim to induce ICD during epigenetic drug therapy, generating highly efficacious cancer immunotherapies.

## Introduction

Antitumor T cells can detect and eliminate cancer cells in a highly precise, antigen-specific fashion. Appropriately activated antitumor T cells can target cancer cells at both local and metastatic sites and, most importantly, can kill existing as well as possibly relapsing cancerous cells. Numerous patient cohort studies thus far have reported a clear positive correlation between the activities of antitumor T cells and better patient outcomes (1–3). Therapeutic interventions promoting antitumor T cell immunity are at the forefront of next-generation cancer therapeutic strategies and as such are highly desired in clinics.

Promoting antitumor T cell responses in cancer-bearing hosts is challenging (4). This is largely because cancers employ numerous evasion strategies that are non-conducive toward T cell activation and function. In particular, cancer-associated immune evasion is supported through the plastic nature of the tumor microenvironment (TME), which harbors the processes that actively suppress the development of antitumor T cells. Some prominent examples of such evasion mechanisms include the presence of immunosuppressive cytokines like transforming growth factor beta 1 (TGF-β) and immune checkpoint molecules such as programmed death-ligand 1 (PD-L1). In addition, immune cells such as myeloid-derived suppressor cells and regulatory T cells contribute to the ability of cancers to evade the immune system (5, 6). Moreover, decreased tumor antigen presentation in the TME further contributes to the impaired functions of antigen-presenting cells (APCs). Consequently, antitumor T cells remain impaired or absent in the immunosuppressive TME, and the tumor persists. Not surprisingly, many modern-day immunotherapies focus on correcting the underlying TME-associated immune evasion strategies, with the goal of facilitating the initiation of an antitumor T cell response (7).

Functional activation of antitumor T cells requires three signals: (#1) tumor antigen presentation in the context of major histocompatibility complex (MHC), (#2) co-stimulatory signals such as cluster of differentiation 28 signaling, and (#3) the presence of cytokines like interferons (IFNs) (8, 9). Although the TME actively discourages the presence of one or more of these essential signals, therapeutic interventions can be used to overcome these immunosuppressive effects. One such strategy is to induce immunogenic cell death (ICD) during cancer therapy (10). As the name suggests, ICD is a process where apoptotic cells elicit an immunogenic response through the induction of damage-associated molecular patterns (DAMPs) that can be recognized by various immune cells (11, 12). More specifically, through the release of DAMPs, ICD increases the adjuvanticity, facilitating the signals # 2 and 3, within TME (10). This occurs through the production of chemoattracting agents such as chemokine C–X–C motif ligand (CXCL) 1 and chemokine (C–C motif) ligand 2 (CCL2) by dying cancer cells and subsequent recruitment of innate immune cells such as neutrophils and DCs to the TME (13). These events, in combination with both the release of nucleic acids from dying cancer cells and a cascade of other DAMPs, enable neo-epitope presentation of the cancer cell (10). This increased antigenicity, facilitating the signal # 1, is reflected through the ICD-enhanced antigen presentation (capture, processing, and presentation *via* MHC) from recruited APCs (10, 13, 14). Consequently, this leads to the activation of T cell response. Importantly, ICD-induced T cell immunity can establish immunological memory capable ensuring the longevity of remission, as opposed to non-regulated cell death. Such processes have been linked to tumor cell death in *in vitro* as well as *in vivo* mouse models (15). Taken together, ICD enhances the adjuvanticity and antigenicity of the cancer cells in the TME and facilitates the development of the three essential signals discussed earlier that are necessary for the activation of antitumor T cell responses (10).

For ICD to be successfully induced, the onset of a specific combination of DAMPs is required. The exact combination of DAMPs needed to induce ICD lies outside of the scope of this review and has been described elsewhere (10, 16). It is important to note, however, that the DAMPs that drive the induction of ICD are dependent on the treatment modality which is being used. For example, while chemotherapy-induced ICD requires the induction of autophagy, pathogen-induced ICD does not (10). Regardless, in the context of ICD, the initiation of an immune response begins with the release of lymphocyte chemoattracting agents, and the presentation of early apoptotic surface markers that tag dying cells for phagocytosis by APCs. In this process, the unfolded protein response (UPR) causes the translocation and expression of endoplasmic reticulum (ER) chaperones, such as calreticulin (CALR), to the cell surface. The induction of autophagy enables the cell to attract APCs to the TME *via* the release of intracellular ATP stores. This further functions to activate both inflammasome signaling and the APCs themselves (17). The secretion of annexin A1 (ANXA1) helps guide the APCs to the dying cancer cells where they become activated. In addition, the extracellular release of high-mobility group box-1 (HMGB1) stimulates an inflammatory response *via* toll-like receptor (TLR)-4 signaling (18). This involves the induction of the type 1 IFN response, resulting in CXCL10 release that enables neutrophil, APC, and T cell recruitment (10, 19, 20). Cumulatively, these ICD hallmarks activate APCs, which then stimulate antitumor T cells, leading to tumor eradication.

Interestingly, the expression of many of these ICD-associated DAMPs is governed by small heritable changes to the genome called epigenetic modifications. Epigenetic modifications result in changes to gene expression through chromatin remodeling mechanisms that include DNA methylation, histone modification, and non-coding RNA (ncRNA) (21, 22). Epigenetic modifications can silence or activate genes involved in tumor suppression or oncogenesis, respectively. In relation to immunity, epigenetic modifying drugs have the potential to boost the immune response by increasing antigen presentation, the expression of co-stimulatory molecules, and the display of MHC molecules; all paving the way for more efficient antigen presentation to T cells (23). In particular, DNA methylation has been investigated in many immune-related studies, where it silences genes such as TLR-3 and mitochondrial–antiviral signaling protein (24–26). Therefore, it is plausible that epigenetic modifications have a regulatory role to play when considering the induction of antitumor immunity.

In this review, we propose that various epigenetic events are actively involved in the regulation of ICD-associated DAMP expression. By recognizing that epigenetic modifications are involved in the induction of individual DAMPs, the efficacy of many cancer immunotherapies can be improved. Herein, we extensively discuss the current evidence that identifies direct links between epigenetic modifications and ICD in the context of TME and cancer immunotherapy.

## Epigenetic Regulation of ICD Hallmarks

**Figure 1:**
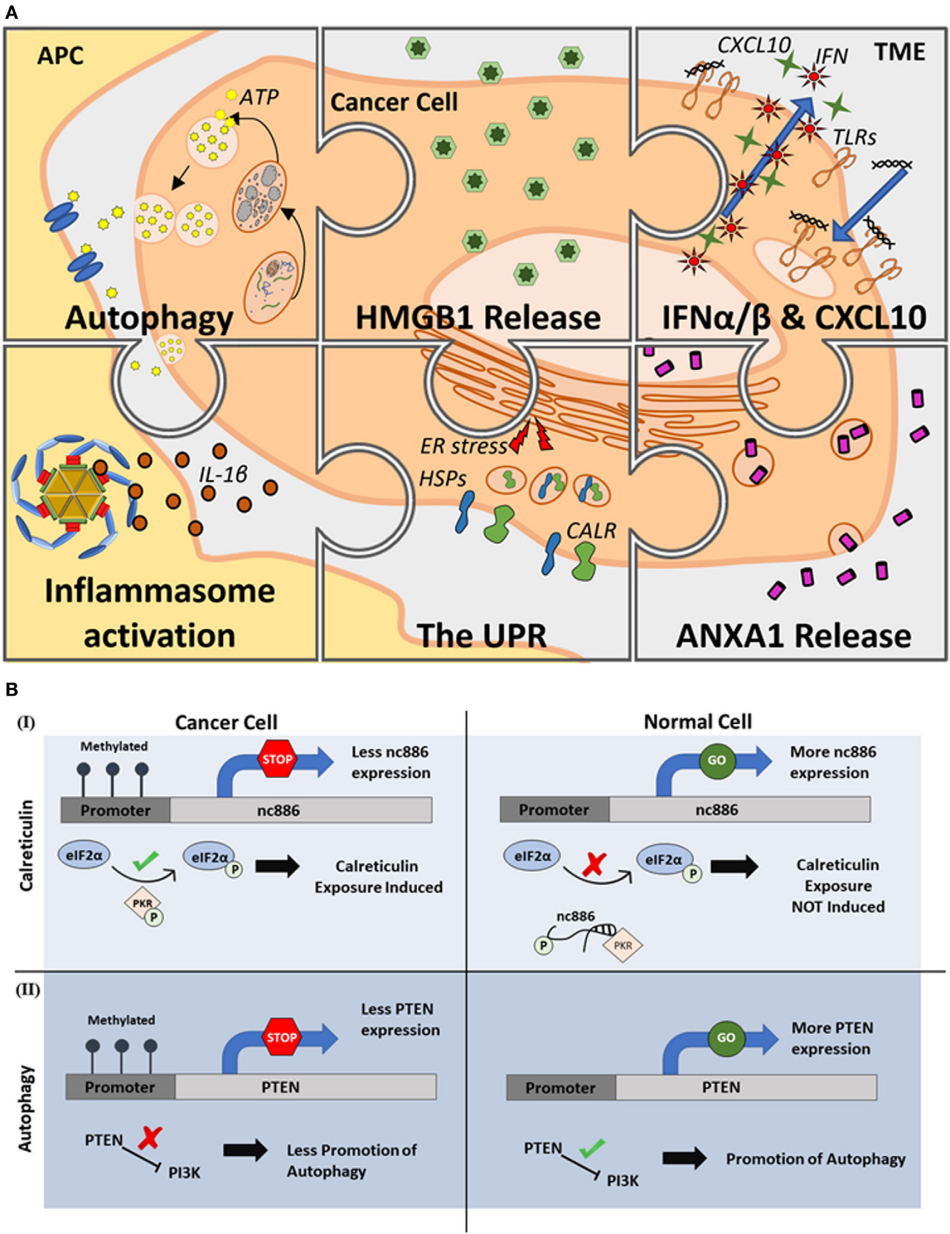
Epigenetic regulation of immunogenic cell death (ICD). **(A)** Major hallmarks of ICD. Induction of ICD has been shown to be associated with six major hallmark processes including the induction of autophagy and release of ATP, high-mobility group box-1 (HMGB1) and annexin A1 (ANXA1) release, toll-like receptor (TLR) signaling that leads to interferon (IFN) α/β and CXCL10 release, inflammasome activation and interleukin-1β (IL-1β) secretion, and endoplasmic reticulum (ER) stress causing the unfolded protein response (UPR) that induces ER chaperones, especially calreticulin (CALR), expression on the cell surface. **(B)** Positive and negative regulation of ICD through epigenetic mechanisms. As illustrated through two distinct examples, activatory (I) or suppressive (II) effects of DNA methylation can either promote or suppress the molecular events leading to ICD. [**(B)**, I], DNA methylation events positively influence the induction of ICD by suppressing the expression of a non-coding RNA (nc886) whose function prevents the successful phosphorylation of eukaryotic transcription initiation factor 2 (eIF2α) inhibiting CALR exposure. [**(B)**, II] DNA methylation events negatively influence the induction of ICD by suppressing the expression of phosphatase and tensin homolog (PTEN) whose expression is needed to initiate pathways leading to autophagy initiation. Abbreviations: APC, antigen-presenting cell; TME, tumor microenvironment.

In the context of cancer therapy, ICD occurs when a therapeutic treatment induces the expression of a specific combination of “hallmarks” during cancer cell death. These hallmarks are a set of premortem stress responses that promote the expression of “danger signals” from the dying cancer cell, which can then be recognized by immune cells to trigger antitumor T cell activation. As shown in Figure 1A, major ICD hallmarks consist of various DAMPs that inevitably result in the development of T cell immunity.

What is becoming increasingly clear is that most of the ICD hallmarks are directly or indirectly regulated through epigenetic mechanisms. In addition, many currently investigated therapeutic epigenetic modulators (e.g., HDAC inhibitors) are being recognized for their actions in dendritic cell activation, antigen uptake, and T cell activation (27, 28) (Table 1). Thus, the epigenome, through its inherent or therapeutically modified activities, can be exploited to harness the antitumor benefits of ICD.

**Table 1:** Epigenetic modulators shown to induce the expression of various ICD hallmarks, studied outside the context of ICD.

Type	Epigenetic modulators	ICD hallmarks							Reference
		UPR	Autophagy	ANXA1	HMGB1	Type I IFN	CXCL10	Inflammasome	
DNMTi	Azacitidine		Yes		Yes	Yes			(29, 30)
	Decitabine					Yes	Yes		(31, 32)
	Zebularine	Yes							(33)

HDACi	Vorinostat (SAHA)						Yes	Yes	(34, 35)
	FR235222			Yes					(36)
	Sodium butyrate			Yes					(37)
	Romidepsin		Yes		Yes		Yes		(30)

HKMTi	Chaetocin	Yes							(38, 39)

*DNMTi, DNA methyltransferase inhibitor; HDACi, histone deacetylase inhibitor; HKMTi, histone lysine methyltransferase inhibitor; UPR, unfolded protein response; ANXA1, annexin A1; HMGB1, high-mobility group box-1; IFN, interferon; CXCL10, C–X–C motif chemokine 10; ICD, immunogenic cell death*.

### The UPR and ER Chaperones

The ER is critically important in the synthesis, modification, and transport of proteins (40, 41). When under physiological stress, the ER initiates the UPR, an evolutionarily conserved mechanism which, in the context of ICD, is characterized by the translocation of ER chaperones to the cell surface (42). Herein, ER chaperones function as “eat me” signals that mark the cell for uptake by APCs (10). Some ER chaperones that have been implicated in ICD include heat shock proteins (HSPs, e.g., HSP70 and HSP90) as well as CALR (43). The UPR is initiated by the activation of three main stress sensors; inositol-requiring enzyme-1 (IRE1), protein kinase RNA-like ER kinase (PERK), and transcription factor 6 (44). The ER chaperone immunoglobin protein (BiP) binds to IRE1 and PERK, suppressing their activity (45). Under ER stress conditions, BiP binds to misfolded proteins and no longer suppresses the sensor’s activity, triggering the UPR (44).

One of the implicated sensors, IRE1, increases in expression upon treatment with an inhibitor of histone lysine methyltransferase (HKMTi). HKMT enzymes work by transferring methyl groups to lysine residues of histone proteins, and in this case, result in the transcriptional silencing of IRE1 (46, 47). Specifically, treatment of lung cancer cells with *Chaetocin* (Table 1), a non-specific HKMTi, increases the expression of IRE1, suggesting that not only is IRE1 regulated *via* BiP but it may also be regulated *via* histone methylation (38, 39).

In relation to the UPR, HSP expression increases in response to stress stimuli as an effort to cope with the denaturation of proteins (48). Two types of HSPs (HSP70 and HSP90) have been shown to be directly regulated *via* promoter methylation in mammalian cells (49, 50).

The distal portion of the promoter region of HSP70 is aberrantly methylated during thermal stress, restricting the binding of POU class 2 homeobox-1 (POU2F1) to the HSP70 promoter (51). In T cells, the role of POU2F1 has been shown to contribute to the timing of cytokine expression in CD4 T cells and has also been shown to promote the development of effector T cell lineages (52). In addition, the expression of HSP90 is inhibited by DNA methylation in both pancreatic and colon cancer cell lines as a consequence of enhanced DNA methyltransferase (DNMT) expression (53). HSP90 regulates the transcription of DNMT enzymes, where HSP90s decrease in expression has been shown to result in an increased expression of DNMTs. DNMT-mediated hypermethylation events then result in the silencing of known tumor suppressor genes (TSGs). For aberrantly methylated HSPs, epigenetic drugs such as Zebularine may correct detrimental hypermethylation events in addition to inducing the UPR helping to induce a more robust immune response (Table 1). Other ER chaperones, such as CALR, are regulated by the presence of ncRNAs whose promoters are hypermethylated in some cancer models (54, 55). In this case, methylation events can work in favor of inducing CALR exposure.

Calreticulin is the most studied “eat me” signal in regards to ICD and is a main player in cultivating the ICD-induced antitumor response (56–58). Although their roles have not been fully elucidated, ncRNA such as RB1 and miR-27a are beginning to be recognized as key players in regulating CALR exposure (54, 55). Within the recent years, however, the roles of a few ncRNA have indeed been more thoroughly analyzed. For example, nc886 has been shown to regulate phosphorylation events that are necessary for proper CALR exposure (Figure 1B) (59). Specifically, eukaryotic transcription initiation factor 2 (eIF2α) is an important protein involved in the exposure of CALR and must be phosphorylated for CALR exposure to be initiated (60). In cholangiocarcinoma, the downregulation nc886 leads to the induction of apoptosis through the phosphorylation of eIF2α (59). When compared with normal tissues, gastric cancer cell lines were found to have hypermethylated CpG islands in the nc886 gene (61). Hypermethylation of nc886 prevents its typical function of discouraging the activation of protein kinase R (PKR), allowing proper phosphorylation of eIF2α and subsequent CALR exposure (Figure 1B). Conversely, expression of nc886 discourages CALR exposure; its expression prevents PKR from catalyzing the phosphorylation of eIF2α, revealing an adverse effect that may be observed with the use of an epigenetic modifying drug. These drugs could further have adverse effects by co-upregulating counterbalancing molecules of ICD hallmarks, such as CD47, a molecule that offsets the pro-phagocytic functions of CALR (62).

#### Autophagy Induction

Autophagy is an evolutionarily conserved mechanism that functions to maintain cellular homeostasis during times of starvation and stress (63). The induction of autophagy enables harmful or damaged cellular components to be sequestered into autophagosomes, and then broken down *via* lysosomal degradation (64). While the role of autophagy in cancer is still being fully elucidated, it appears to be context dependent (65, 66).

During the process of ICD, the induction of autophagy results in vesicular ATP pools to be transported and secreted from the cell (10). The secretion of ATP activates signaling pathways *via* purinergic receptors P2Y2 (P2RY2) and P2RX7 acting as a “find me” signals that promote maturation as well as TME recruitment of APCs (19, 20, 67). In APCs, the interaction of ATP with P2RY2 induces a robust chemotactic effect, while its interaction with P2RX7 results in the release of immunostimulatory cytokines (67). The expression of the P2RX7 receptor has been shown to be controlled *via* promoter methylation in submandibular carcinomas, where aberrant methylation events decrease its expression, which presumably would prevent proper P2RX7 signaling during ICD (68).

It is important to note that while other mechanisms are capable of triggering ATP release (69–71), a successful autophagic response is required for the optimal levels of ATP to be released for an immunogenic response (10, 64, 67). Autophagy induction requires multiple cellular processes to occur in tandem, such as the expression of TSGs phosphatase, tensin homolog (PTEN), and autophagy-related protein 5 (ATG5) (10). PTEN promotes autophagy by inhibiting the activation of phosphoinositide 3-kinase (PI3K) signaling (67) (Figure 1B), while ATG5 mediates autophagosome formation (72). Interestingly, PTEN is one of the most commonly mutated or inactivated genes during cancer development (73). The PTEN promoter is also known to be hypermethylated in breast and gastric cancers, as well as in melanoma and soft tissue sarcomas (74–77). During the development of many cancers, including colorectal cancer and melanoma, ATG5 is often downregulated (78, 79). Interestingly, it has been demonstrated in melanoma that ATG5 downregulation is a consequence of a hypermethylation of the promoter site (79). The hypermethylation status of these genes in cancers represents an ideal target for demethylating agents (e.g., Azacitidine) to promote autophagy (Table 1).

It is possible to induce autophagy through many mechanisms. Caloric restriction mimetics (CRMs), which induce autophagy by mimicking biochemical effects of nutrient deprivation, have been shown to stimulate ATP release in a protein deacetylation-dependent manner. Specifically, CRMs influence the acetylation of histone proteins, ultimately influencing gene expression and displaying a potential epigenetic mechanism that influences whether or not autophagy is induced (32, 80). Related, autophagy can also be induced *via* photodynamic therapy. Following exposure to photosensitizers, multiple human cancer cell lines showed the surface expression of CALR and released ATP before the signatures of apoptosis could be detected. In fact, both of these processes seem to have overlapping regulatory mechanisms, operating through PERK signaling and PI3K pathways, suggesting that the interplay between ICD and DAMP induction requires further elucidation (58).

#### ANXA1 Release

Annexin A1, known for its immunosuppressive functions (81), has recently been found to contribute to DC function during ICD. Here, ANXA1 released from the apoptotic cells can bind to formyl peptide receptor 1 receptor on APCs, enabling the stable interaction between the APC and dying cancer cell (82–84). As such, ANXA1 functions to enable antigen uptake and cross presentation of tumor antigens (84). Interestingly, ANXA1 is silenced by methylation in nasopharyngeal cancer cell lines and aberrantly methylated in breast and non-small cell lung cancer (85–88). Here, the use of a DNMTi may be an attractive tool, allowing the restoration of ANXA1 expression and secretion in the context of ICD (Table 1).

In head and neck squamous carcinoma, the expression of ANXA1 is inversely correlated with the expression of a specific microRNA (miRNA-196). This miRNA directly targets ANXA1 by binding to the untranslated region on the ANXA1 mRNA transcript (82). The expression of this miRNA is controlled by DNA methylation in many different cancer cell lines including breast, colon, liver, lung, brain, and oral (89). Without the expression of miRNA-196, ANXA1 would no longer be silenced, allowing proper ANXA1 release during ICD induction. As mentioned, epigenetic modifiers (either hypo- or hypermethylating) can be employed in the context of cancers and TME. In this case, induction of *de novo* DNA hypermethylation by inserting CpG-free DNA may control the miRNA-196-regulated release of ANXA1 for ICD induction (90).

#### HMGB1 Release

High-mobility group box-1 is found in nearly all eukaryotic cells and is highly conserved and abundant (91). Much like autophagy, it is important to note that HMGB1 has both a positive and negative correlation in regard to cancer progression (92). HMGB1 has multiple roles: within the nucleus it facilitates the transcription of many genes by modulating nucleosomes, while when secreted, it functions as a DAMP (91, 93). The mechanisms that regulate this secretion, however, remain unclear (10, 94). Extracellular HMGB1 signaling is facilitated by numerous receptors, where its binding is heavily dependent on its redox form (94). An important signaling pathway in the context of ICD is the extracellular binding of HMGB1 to TLR4 on APCs, initiating a signal transduction through the adaptor protein MyD88 (10, 18, 94). This pathway has been shown to be required to evoke ICD and subsequent T cell immunity, as Tlr4^−/−^ and Myd88^−/−^ mice do not develop antitumor immunological memory (18).

The link between HMGB1 and epigenetic regulation has already been hypothesized, and it is postulated that HMGB1 itself acts as an epigenetic modifier that leads to the silencing of tumor necrosis factor alpha and interleukin-1 beta (IL-1β) (91). Like the miRNA-196-based regulatory mechanism discussed in relation to ANXA1 release, miRNA-129-2, a tumor suppressor in glioma and hepatocellular carcinoma (95, 96), directly targets HMGB1 and inhibits its release. The regulatory region of this miRNA is heavily methylated in portions of its promoter region, resulting in its suppression and subsequent expression of HMGB1 (97, 98). In relation to gliomas, this methylation occurs more frequently in cancerous tissues when compared with normal tissues (95). As with some of the previously discussed ICD hallmarks, this implication is positive in relation to ICD. Interestingly, inducing expression of this miRNA would not be beneficial in this case. In fact, similar to ANXA1, the induction of *de novo* methylation in models where this miRNA is expressed may be a better choice of treatment (90).

#### Type I IFN Production and CXCL10 Secretion

Type I IFNs are secreted as DAMPs from infected cells to both signal and activate antimicrobial responses and initiate the innate and adaptive immune system (10). Characteristic type I IFNs (IFNα and IFNβ) primarily signal through the heterodimeric IFNα receptor (10, 99), eliciting a vast range of responses that are dependent on environmental factors, the extent of infection, and the hosts’ physiological status (99). In the context of ICD, a major role for type I IFNs is to activate signaling cascades to produce more IFNs that act in both an autocrine and paracrine fashion (10). Moreover, like ATP secretion, type I IFNs also act as chemokines to attract APCs to the TME and play a pivotal role in APC maturation and T cell activation (100, 101). Thus, type I IFNs are important mediators of the signals # 2 (co-stimulatory signals) and 3 (cytokine presence) that are required for the induction of T cell immunity.

A clear link exists between epigenetic regulation and IFNs. First, expression of HDAC3 (a histone deacetylase) has been found to be necessary for IFN-β expression showing the regulatory role of HDAC3 in controlling IFN-β expression (99). Second, CXCL10 secretion is a subsequent result of IFN signaling (102). Its expression has been found to increase upon treatment with demethylating agents in ovarian cancer cells, suggesting that promoter methylation controls CXCL10 expression (103). The epigenetic regulation of CXCL10 in ovarian cancer suggests that treatment with a demethylating agent such as Decitabine (which is also known to induce type I IFN signaling) could aid in the induction of ICD (Table 1).

#### NLRP3 Inflammasome Signaling

During ICD induction, DAMPs are able to trigger pro-inflammatory events by activating inflammasomes (104). Inflammasomes are large multi-protein complexes, often consisting of caspase 1, within which the maturation of pro-inflammatory cytokines such as IL-1β and IL-18 takes place (104). One of the most well-characterized inflammasome complexes is called NLR family pyrin domain containing 3 inflammasome (NLPR3), which consists of a caspase recruiting domain (ASC), a cytosolic pattern recognition receptor, and a pro-caspase 1 (105). In 2014, Salminen et al. found that the ASC domain is identical to the domain that was termed as methylation-induced silencing-1 (TMS1) (105). The same study outlined that the promoter of the TSM1 gene is aberrantly methylated in many cancer cell lines, suggesting that this process regulates the expression of inflammasomes and the induction of apoptosis. Assuming methylation events alter the function of the ASC domain, aberrant events may prevent the successful induction of ICD by preventing proper inflammasome formation.

Another layer of complexity is added when the production of IL-1β is considered. Many interleukin genes have been shown to be methylated in cancers (106–108). In addition, interleukins have also been shown to have powerful antitumor roles by inhibiting the growth of lung tumors, and by stimulating the immune system to engage antiangiogenic mechanisms (109). Interestingly, the promoter of IL-1β has the highest methylation status out of all interleukin genes studied in lung cancer (29). In this model, aberrant promoter methylation of this important ICD-related interleukin would prevent the successful induction of ICD, revealing a potential therapeutic target using a demethylating drug (Table 1).

## Conclusion and Future Directions

The immunogenic response initiated through ICD can overcome the immunosuppressive nature of the TME. This leads to the restoration of the three signals required for proper T cell activation, including increased antigen presentation following cancer cell apoptosis and phagocytosis (signal # 1), co-stimulation from matured and recruited APCs (signal # 2), and the production of cytokines from both the cancer (e.g., IFNs) and APCs (e.g., IL-1β) (signal # 3). Therefore, the successful induction of ICD leads to the activation of antitumor T cells, which in turn can kill cancer cells and prevent recurring disease. Therefore, understanding how epigenetic modifications contribute to ICD is important when aiming to improve the efficacy of current cancer immunotherapies.

As highlighted above, many of the processes that govern ICD are regulated through epigenetic modifications. Interestingly, the initiation of individual ICD hallmarks, upon treatment with epigenetic modifying drugs, has been observed in studies that may not have been directly evaluating ICD induction (Table 1). As a result, the combination of epigenetic modifiers and immunotherapies offer an attractive avenue to elicit more robust antitumor T cell immunity. In fact, this concept is already being applied. The combination of Azacitidine and Romidepsin with IFNα elicits *bona fide* ICD in colorectal cancer cells (30). Further, treatment with Decitabine triggers a “viral” or “altered-self” mimicry state in these cells that leads to ICD hallmark expression through the retinoic acid inducible gene-I (RIG-I) pathway (31). This pathway has been shown to evoke ICD in melanoma, acute-promyelocytic leukemia, and pancreatic cancer models (31). Most recently, this concept has been shown to be important in neutrophil-based anticancer activity, where apoptotic cancer cells release epigenetically regulated cytokines such as CXCL1, CXCL10, and CCL2, driving nucleic acid-elicited phagocytosis of dying cancer cells by neutrophils (13, 110, 111).

However, it is still important to consider the possibility that epigenetic modifications could negatively affect the ability of CD8 T cells to recognize a cancer cell through ICD. It has already been established that epigenetic mechanisms tightly regulate the expression of MHC molecules, cytokines and other co-stimulatory molecules (112). Therefore, it cannot be ignored that adjusting these regulatory pathways using epigenetic modifiers may reduce the successful activation of specific CD8 T cells. In addition, increasing the secretion of a desired DAMP using epigenetic modulators may affect the expression of checkpoint molecules such as PD-L1 or suppressive metabolites such as indoleamine 2,3-dioxygenase (IDO1) in the cancer cells, causing them to respond to immunotherapeutic strategies differently. It has also been established that the miRNA-regulated mechanisms that control the expression of PD-L1 are also involved in the repression of IDO1 in cancer cells (113, 114). These points stress the complex relationship that exists between using epigenetic modifiers and their effect on ICD DAMPs.

Finally, the context-dependent roles of DAMPs must also be noted while considering ICD-based implications. For example, while HMGB1 excretion is involved in DC-based nucleic acid-sensing systems in ICD (115), it has also been shown to silence the expression of IL-1β in severe systemic inflammation by binding with histone H1, causing a change from euchromatin to heterochromatin at the IL-1β promoter (116). Therefore, the induction of one process (e.g., autophagy) that regulates a hallmark may suppress another (e.g., CALR exposure). Thus, when aiming to improve cancer therapy using epigenetic modifiers to induce hallmarks of ICD, the methylation status of ICD-related genes should be analyzed in each cancer model. This will enable an evaluation of both the benefits and adverse events that could result from the treatment modality of interest. Nonetheless, there is an undeniable link between the regulation of ICD hallmarks and epigenetics that cannot be ignored when evaluating the efficacy of novel cancer treatments.

## Author Contributions

BC and MG: conception, research, writing, editing. SG: conception, research, writing, editing, funding. PM, JP, and SM: research, writing, editing.

## Conflict of Interest Statement

The authors declare that the research was conducted in the absence of any commercial or financial relationships that could be construed as a potential conflict of interest.
